# Ultra-low concentration ether electrolytes with strong Coulomb interactions for high-voltage lithium metal batteries†

**DOI:** 10.1039/d4sc07393b

**Published:** 2025-03-25

**Authors:** Chengkun Liu, Zhipeng Jiang, Yuhang Zhang, Wenjun Xie, Jiahang Zou, Shilin Wu, Mengjun Sun, Yongtao Li

**Affiliations:** a School of Materials Science and Engineering, Anhui University of Technology Maanshan 243002 China jzp1994@ahut.edu.cn liyongtao@ahut.edu.cn; b School of Chemistry and Chemical Engineering, Henan Normal University Xinxiang 453007 China sunmengjun@htu.edu.cn; c Key Laboratory of Efficient Conversion and Solid-state Storage of Hydrogen & Electricity of Anhui Province Maanshan 243002 China

## Abstract

The advancement of high-energy-density lithium metal batteries (LMBs) necessitates the development of novel electrolytes capable of withstanding high voltages. Ether-based electrolytes, while compatible with lithium metal anodes (LMAs), face limitations in high-voltage stability. Traditional design strategies with high concentration enhance the high-voltage stability of electrolytes by consuming free solvents to prevent their decomposition but face high-cost issues. Herein, we introduce a novel design approach for high-voltage ether electrolytes that leverages strong Coulomb interactions between lithium ions (Li^+^) and anions to construct an anion-dominated solvation structure. This solvation structure not only enhances de-solvation kinetics but also forms stable anion-derived interfaces at both electrodes, thereby maintaining electrode stability and preventing free solvent decomposition. Li-LiNi_0.8_Co_0.1_Mn_0.1_O_2_ (NCM811) cells using a strong Coulomb force electrolyte (SCE) designed based on this principle demonstrate superior rate performance (20C/120.8 mA h g^−1^) and cycling stability (5C/1000 cycles). Notably, even at an ultra-low concentration of 0.1 M, Li-NCM811 cells utilizing the SCE exhibit good rate performance (5C/121.9 mA h g^−1^) and stable cycling over 200 cycles at a cutoff voltage of 4.4 V. This approach provides a high-performance and cost-effective electrolyte solution for practical high-voltage LMB applications.

## Abstract 2

An ultra-low concentration ether electrolyte for high-voltage LMBs creates an anion-dominated solvation structure *via* strong Li^+^–anion interactions. This structure enhances de-solvation kinetics and forms stable interfaces on both electrodes.

## Graphical abstract

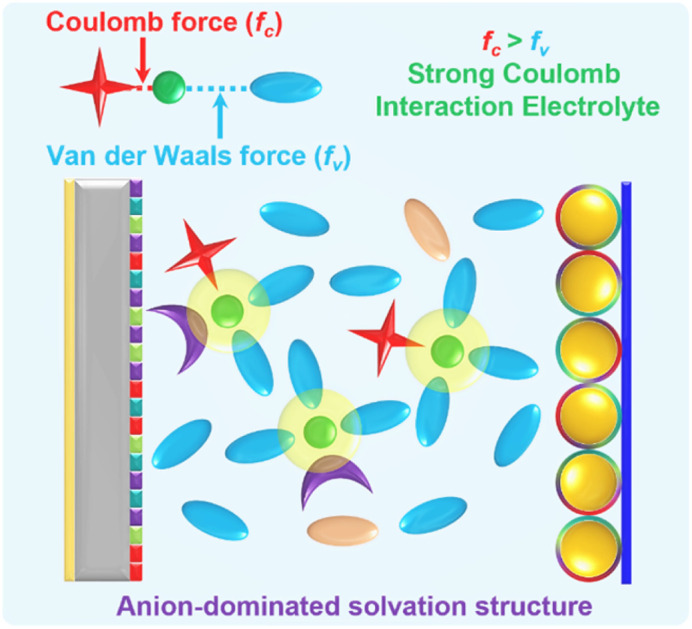

## Introduction

High-voltage lithium metal batteries (LMBs) promise significant energy density improvements over lithium-ion batteries (LIBs), making them a prime candidate for next-generation energy storage systems.^1^ However, the commercialization of high-voltage LMBs is limited by their short cycle life and high costs.^2^ The instability of the lithium metal anode (LMA) and the structural degradation of high-voltage cathodes are primary factors contributing to poor cycle performance.^3^ Enhancing the stability of the electrode–electrolyte interphase through electrolyte engineering is critical to addressing these challenges.^4^ 1,2-Dimethoxyethane (DME) is widely used in LMB electrolytes due to its excellent compatibility with lithium metal anodes (LMA).^5–7^ However, its decomposition at high voltages (>4.0 V) limits its use with various high-voltage cathodes, including LiNi_1−*x*−*y*_Co_*x*_Mn_*y*_O_2_ (NCM), LiNi_1−*x*−*y*_Co_*x*_Al_*y*_O_2_ (NCA), and LiCoO_2_ (LCO).^8–10^ High-concentration electrolytes (HCEs), with Li salt concentrations exceeding 3 M, mitigate this issue by reducing the number of free solvent molecules, thereby enhancing the high-voltage stability of DME.^11,12^ Nevertheless, the high viscosity and poor wettability of HCEs restrict their applicability.^13^ Zhang *et al.* designed localized high-concentration electrolytes (LHCEs) by incorporating non-solvating co-solvents, with Li salt concentrations of 1–2 M.^14,15^ This approach preserves the “solvent-in-salt” solvation structure, allowing DME to be paired with various high-voltage cathodes operating above 4.3 V while improving physicochemical properties.^16–18^ In our previous work, we found that using LiDFOB as the primary salt can alter the decomposition pathway of DME at the cathode, enabling high-voltage stability in DME-based electrolytes at a conventional concentration of 1.5 M without the addition of diluents.^19–21^ However, these electrolytes do not offer significant cost advantages and still face challenges for large-scale applications. Reducing Li salt concentration is an effective way to lower electrolyte costs.^22–26^ Based on this consideration, there is an urgent need to develop a low-concentration, high-voltage electrolyte to meet the practical requirements of LMBs.

**Fig. 1:**
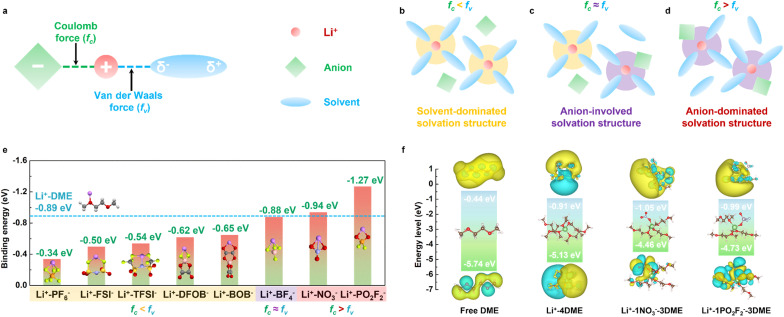
(a) Schematic diagram of the microscopic forces between Li^+^, anions and solvent molecules. (b–d) Schematic diagram of different types of solvation structures. (b) Conventional electrolyte, (c) WSE, and (d) SCE. (e) Comparison of the binding energy of Li^+^ with DME molecules and different anions. (f) Comparison of LUMO–HOMO energy levels of typical solvation structures.

We propose a new design principle for high-voltage electrolytes. By modulating the strength of microscopic forces between lithium ions (Li^+^), anions, and solvent molecules, we can further regulate the solvation structure of the electrolyte. As shown in Fig. 1a, in electrolyte solutions, the interaction between Li^+^ and anions mainly occurs through Coulomb forces (*f*_c_), while the interaction between Li^+^ and solvent molecules primarily involves van der Waals forces (*f*_v_). The relative strengths of Coulomb forces and van der Waals forces determine the solvation structure of the electrolyte. In conventional electrolytes, *f*_c_ is usually smaller than *f*_v_, leading to a solvent-dominated solvation structure where a large number of solvent molecules occupy the first solvation sheath (Fig. 1b).^27^ Using a solvent with a weaker solvating ability, such as dipropyl ether (DPE)^28^ or tetrahydropyran (THP)^29^ instead of DME to construct a weakly solvating electrolyte (WSE),^30^ has been shown to be an effective electrolyte optimization strategy. This approach reduces *f*_v_, making it approximately equal to *f*_c_, allowing anions to partially enter the first solvation sheath (Fig. 1c). This solvation structure can increase the inorganic content at the interface, thereby improving the performance of LMBs.^31^

Herein, we designed a strong Coulomb force electrolyte (SCE) with a specific composition of 0.25 M LiNO_3_ + 0.25 M LiPO_2_F_2_ in DME/FEC (8 : 2, v/v), successfully achieving high-voltage stability of DME-based ether electrolytes at ultra-low concentrations. In this electrolyte, *f*_c_ is greater than *f*_v_, which can result in an anion-dominated solvation structure (Fig. 1d). Unlike HCEs and LHCEs, SCE exhibits excellent high-voltage stability even though a large amount of DME exists as free solvent. This is because the anion-dominated solvation structure can form an anion-derived stable interface on both the cathode and the anode, simultaneously suppressing DME decomposition at the cathode and providing high cycling stability for the LMA. Electrochemical tests show that SCE enables Li-LiNi_0.8_Co_0.1_Mn_0.1_O_2_ (NCM811) cells to achieve stable charge–discharge performance at 20C with a high discharge capacity of over 120 mA h g^−1^ under a cut-off voltage of 4.4 V, and stable cycling over 1000 cycles at 5C with a capacity retention of 76.2%. Even under practical conditions, SCE can achieve stable cycling of Li-NCM811 full cells (8.3 mg cm^−2^, 50 μm Li) for over 500 cycles. In addition, the commercial Cu-NCM811 anode-free cell (cathode loading of 18 mg cm^−2^) assembled based on SCE can also cycle stably more than 60 times with an average Coulombic efficiency (CE) of 97.5%. More importantly, we further reduced the Li salt concentration to 0.1 M based on this electrolyte design principle. As a result, we achieved compatibility of a 0.1 M ultra-low concentration DME-based electrolyte with 4.4 V Li-NCM811 cells without using any diluents, enabling stable cycling for over 200 cycles at 2C.

## Results and discussion

To design an electrolyte with strong Coulomb interactions, we conducted density functional theory (DFT) calculations to assess the binding energies between Li^+^ and various anions, as well as between Li^+^ and the DME solvent, as illustrated in Fig. 1e. The calculations reveal that the binding energy of Li^+^ with DME is −0.89 eV, which surpasses the binding energies of Li^+^ with several anions, including PF_6_^−^, FSI^−^, TFSI^−^, DFOB^−^, and BOB^−^, which are −0.34 eV, −0.50 eV, −0.54 eV, −0.62 eV, and −0.65 eV, respectively. This suggests that these Li salts are more likely to fully dissociate in DME, leading to a solvent-dominated solvation structure, consistent with previous literature. The binding energy between Li^+^ and BF_4_^−^ is −0.88 eV, which is similar to the binding energy of Li^+^ with DME. Therefore, utilizing LiBF_4_ as the primary salt facilitates the formation of an anion-regulated weakly solvating electrolyte, as demonstrated in our earlier work.^32^ It is noteworthy that, unlike conventional Li salts, LiNO_3_ and LiPO_2_F_2_ exhibit strong Coulomb interactions, with binding energies of −0.94 eV and −1.27 eV, respectively, both of which are higher than the van der Waals interactions present between Li^+^ and DME. Thus, using LiNO_3_ and LiPO_2_F_2_ as the primary salts can produce an anion-dominated solvation structure. To elucidate the solvation environment of Li^+^ in various electrolyte systems, we conducted ^7^Li nuclear magnetic resonance (NMR) spectroscopy in a DME/FEC (8 : 2 v/v) solvent system. As illustrated in Fig. S1,† highly dissociative salts such as LiPF_6_ and LiFSI exhibit chemical shifts of −1.275 ppm and −1.254 ppm, respectively, whereas LiNO_3_ and LiPO_2_F_2_ display significantly less negative shifts at −0.033 ppm and −0.343 ppm. This substantial difference indicates that LiNO_3_ and LiPO_2_F_2_ enhance anion coordination with Li^+^, leading to a pronounced deshielding effect that counteracts the solvent-induced shielding. The impact of this solvation behavior is further corroborated by ionic conductivity measurements, which demonstrate that LiNO_3_ and LiPO_2_F_2_-based solutions exhibit markedly lower room-temperature conductivity than LiPF_6_ and LiFSI-based solutions, consistent with their lower degree of ionic dissociation (Fig. S2†). These experimental observations strongly align with theoretical predictions, confirming that LiNO_3_ and LiPO_2_F_2_ exhibit a significantly higher *f*_c_ relative to *f*_v_, thereby promoting the formation of an anion-rich inner solvation sheath that affects the electrolyte's ionic transport properties. Notably, due to the insolubility of LiPF_6_ in pure DME, as shown in Fig. S3,† we chose to add a small amount of FEC (DME/FEC, 8 : 2 v/v) to promote the dissolution of LiPF_6_ for subsequent electrochemical testing. Furthermore, we performed DFT calculations on the lowest unoccupied molecular orbital (LUMO) and highest occupied molecular orbital (HOMO) energy levels of pure DME, DME-dominated solvation structures (Li^+^-4DME), NO_3_^−^-involving solvation structures (Li^+^-1NO_3_^−^-3DME), and PO_2_F_2_^−^-involving solvation structures (Li^+^-1PO_2_F_2_^−^-3DME) to reflect the oxidation and reduction decomposition ability of different solvation structures at the electrodes. As shown in Fig. 1f, compared to pure DME, solvated DME exhibits lower LUMO and higher HOMO energy levels, indicating that solvated DME is more prone to decomposition, resulting in a large amount of organic components at the interface. However, the introduction of NO_3_^−^ and PO_2_F_2_^−^ into the first solvation sheath significantly reduces the LUMO and HOMO energy levels of the solvation structure, indicating that these components decompose preferentially over DME at the anode and cathode interfaces, producing a large amount of inorganic-rich cathode electrolyte interphase (CEI) and solid electrolyte interphase (SEI), thus providing effective protection for LMBs.^33^ Based on this design principle, we designed a strong Coulomb force electrolyte for subsequent characterization and testing, with the specific composition of 0.25 M LiNO_3_ + 0.25 M LiPO_2_F_2_ in DME/FEC (8 : 2, v/v). In contrast, we also designed a weak Coulomb force electrolyte (WCE) as a control to validate the effectiveness of this approach, with the specific composition of 0.25 M LiPF_6_ + 0.25 M LiFSI in DME/FEC (8 : 2, v/v). Additionally, we used a conventional carbonate electrolyte (CCE, 1 M LiPF_6_ in EC/DEC/FEC (4 : 4 : 2, v/v)) with a Li salt concentration of 1 M as a control group to highlight the superiority of SCE.

**Fig. 2:**
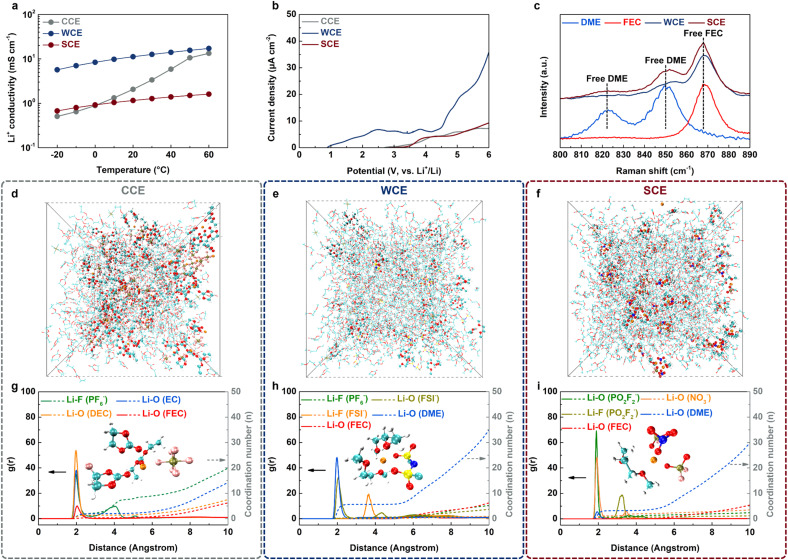
(a) The Li^+^ conductivity comparison of studied electrolytes at various temperatures. (b) LSV plots of studied electrolytes. (c) Locally amplified Raman spectra for different solutions. (d–f) Snapshots of the MD simulation cell and (g–i) the corresponding radial distribution functions of different electrolytes: (d) and (g) CCE, (e) and (h) WCE, (f) and (i) SCE. The insets show the representative Li^+^ solvation structures of the electrolytes.

The conductivity results of electrolytes at different temperatures reveal that although DME has strong solvation abilities, the stronger Coulomb interactions between Li^+^ and anions in SCE lead to a lower room-temperature conductivity compared to CCE (Fig. 2a). In contrast, WCE exhibits significantly higher conductivity than CCE. At low temperatures, the high freezing point of carbonate electrolyte results in a substantial drop in conductivity, making it lower than that of SCE.^34^ The strong Coulomb interactions in SCE also confer superior high-voltage stability, as linear sweep voltammetry (LSV) results show that SCE maintains high oxidation resistance, similar to CCE, without a significant increase in current density even at voltages up to 6 V (Fig. 2b). In contrast, WCE exhibits a marked increase in current density at 4.2 V, indicating substantial electrolyte decomposition. Raman spectroscopy comparison shows that SCE contains more free solvents than WCE, suggesting that DME in WCE is more likely to form a solvent-dominated solvation structure by entering the first solvation shell (Fig. 2c and S4†). Conversely, the first solvation shell in SCE is mainly occupied by anions, with DME pushed out into the outer shell as free solvent. This solvation structure is opposite to that of HCEs and LHCEs, which tend to consume rather than expel free solvents.^35–37^ Molecular dynamics (MD) simulations offer further insight into the solvation structures and interactions at the microscopic level (Fig. 2d–f). Radial distribution function (RDF) analysis confirms that in CCE and WCE, DEC and DME molecules, respectively, dominate the first solvation shell (Fig. 2g–h). In contrast, the first solvation shell in SCE is mainly composed of PO_2_F_2_^−^ and NO_3_^−^ anions, with only minor amounts of DME present, highlighting the formation of an anion-dominated solvation structure due to strong Coulomb interactions between Li^+^ and the anions (Fig. 2i). Additional calculations of the solvation structures for 0.5 M LiNO_3_ and 0.5 M LiPO_2_F_2_ single salts in the same solvent system (DME : FEC = 8 : 2) indicate a similar anion-dominated structure, underscoring that this unique solvation structure in SCE stems from intrinsic Li^+^–anion interactions rather than the mixed addition of anions (Fig. S5 and S6†). Notably, FEC enters the first solvation shell only in CCE, where it participates in Li salt dissociation, while in other electrolytes, FEC remains as free solvent, showing that FEC does not significantly influence the interactions between Li^+^, anions, and DME.

**Fig. 3:**
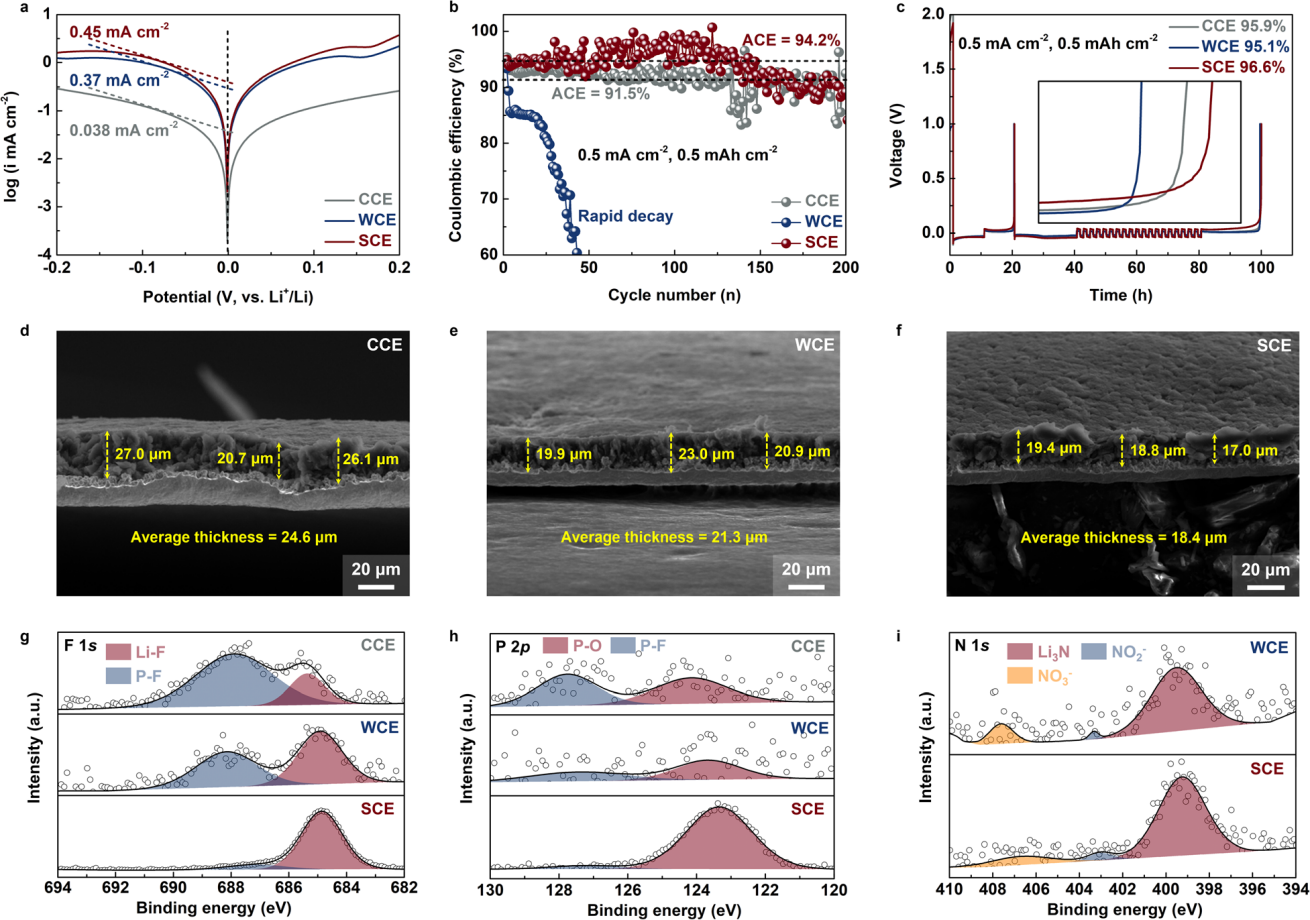
(a) Tafel plots of Li–Li symmetric cells using studied electrolytes. Li–Cu half-cell (b) cycling and (c) CE tests using different electrolytes (test conditions: 0.5 mA cm^−2^, 0.5 mA h cm^−2^ for 20 cycles). SEM cross-sectional images of deposited Li using (d) CCE, (e) WCE, and (f) SCE (deposition conditions: 0.5 mA cm^−2^ for 3 mA h cm^−2^). (g) XPS F 1s, (h) P 2p, and (i) N 1s spectra of metallic Li deposited from different electrolytes.

We conducted a comprehensive study on the influence of the aforementioned electrolytes on LMAs. Tafel tests revealed that SCE exhibited the highest exchange current density of 0.45 mA cm^−2^ compared to the others, indicating superior Li^+^ deposition kinetics (Fig. 3a).^38^ In Li–Cu half-cell tests under conditions of 0.5 mA cm^−2^ and 0.5 mA h cm^−2^, WCE exhibited a significant drop in CE after fewer than 30 cycles, whereas SCE remained stable for over 200 cycles, with an average coulombic efficiency (ACE) of 94.2%, outperforming CCE's 91.5% (Fig. 3b).^39^ Furthermore, using Adams' method, we accurately measured the CE of the LMA in the three electrolytes.^40^ The results corroborated that metallic Li in SCE also had the highest deposition reversibility, with a CE of 96.6%, compared to 95.9% in CCE and 95.1% in WCE (Fig. 3c). Optimization studies further showed that the LiNO_3_–LiPO_2_F_2_ dual-salt system provided better cycling reversibility than the single-salt systems, and the optimal concentration ratio was determined to be 1 : 1, likely due to differences in the composition of the formed interface (Fig. S7 and S8†).^41^ The ideal DME–FEC ratio was determined to be 8 : 2, as too much FEC could disrupt interactions between Li^+^, anions, and DME, while the absence of FEC could be detrimental to the formation of a stable SEI (Fig. S9†). Morphological analysis *via* SEM cross-sections revealed that, at a deposition capacity of 3 mA h cm^−2^, the average Li deposition thickness in CCE, WCE, and SCE was 24.6 μm, 21.3 μm, and 18.4 μm, respectively. This result indicates that metallic Li in SCE tends to form large and dense grains, which can be further corroborated by SEM top-view images (Fig. 3d–f and S10–S12†).^42^ This morphology formation is intricately linked to the SEI composition on the Li surface. XPS elemental analysis revealed that, despite the Li salt concentration being only 0.5 M in SCE, the content of anion-derived inorganic components (closely related to the elemental contents of F, P, and N) significantly exceeded those in CCE and WCE, suggesting that strong Coulomb interactions facilitate the formation of anion-derived SEI layers (Fig. S13†). Specifically, the XPS F 1s spectra indicated that LiPF_6_ in CCE and WCE did not fully decompose to produce LiF (685.4 eV) but generated significant by-products containing P–F bonds (687.9 eV, Fig. 3g).^43^ Conversely, the SEI formed in SCE was rich in LiF, primarily derived from LiPO_2_F_2_ decomposition, with some contributions from FEC. The XPS P 2p spectrum further supported this, showing that the P in the SEI formed in SCE mainly existed as P–O bonds (Li_2_PO_4_, 134.2 eV), while CCE and WCE showed the presence of by-products with P–F bonds (137.8 eV, Fig. 3h).^44^ Li_2_PO_4_ has been documented to provide effective protection for LMAs. Additionally, the XPS N 1s spectrum revealed that, although both WCE and SCE introduced N-rich anions (FSI^−^ and NO_3_^−^), due to strong Coulomb interactions with Li^+^, NO_3_^−^ in SCE preferentially decomposed to generate a substantial amount of Li_3_N (399.4 eV), which facilitates rapid Li^+^ transport within the SEI (Fig. 3i).^45^

**Fig. 4:**
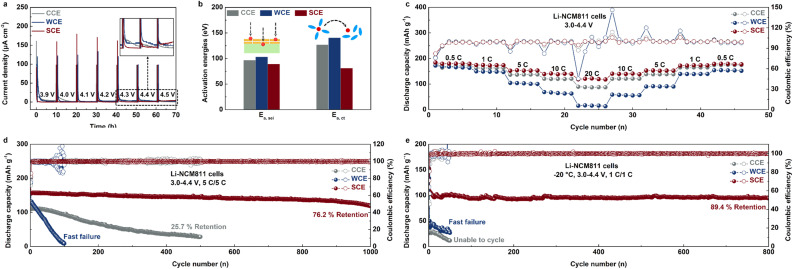
(a) Potentiostatic tests of Li-NCM811 cells using studied electrolytes. (b) Comparison of the activation energy of different electrolytes calculated using the Arrhenius equation. (c) Rate, (d) room-temperature long-term cycling, and (e) low-temperature long-term cycling tests of Li-NCM811 cells using different electrolytes (test conditions are 0.5C, 1C, 5C, 10C, 20C, 5C/5C at 25 °C, and 1C/1C at −20 °C, respectively; 3.0–4.4 V, 1C = 180 mA g^−1^).

To explore the compatibility of novel electrolytes with high-voltage NCM811 cathodes, we conducted potentiostatic testing on Li-NCM811 cells (Fig. 4a).^46^ Remarkably, the SCE electrolyte demonstrated the lowest and most stable constant current response at cutoff voltages of 4.3 V and 4.4 V, in contrast to both WCE and CCE, underscoring its potential for high-voltage LMB applications. Electrochemical impedance spectroscopy (EIS) on Li–Li symmetric cells across different temperatures enabled the calculation of interfacial resistance (*R*_sei_) and charge transfer resistance (*R*_ct_, Fig. S14†). Fitting these results to the Arrhenius equation provided activation energies (*E*_a,sei_ and *E*_a,ct_), which indicate the efficiency of Li^+^ transport and Li^+^ de-solvation ability at the interface (Fig. 4b and S15†).^47^ The data demonstrated that the SCE-derived SEI layer exhibited superior Li^+^ transport, with the lowest *E*_a,sei_ value. This may be due to the ability of SCE to decompose, producing Li_3_N and Li_3_PO_4_ with high ionic conductivity, which significantly enhances Li^+^ transport behavior at the interface.^48,49^ Additionally, the strong Coulomb interactions between Li^+^ and anions facilitated rapid de-solvation at the interface, leading to the lowest *E*_a,ct_ value. These advantageous interfacial properties enabled the Li-NCM811 cell using SCE to deliver excellent rate performance. At a cutoff voltage of 4.4 V, the discharge capacities were 180.0 mA h g^−1^, 174.3 mA h g^−1^, 154.0 mA h g^−1^, 141.4 mA h g^−1^, and 120.8 mA h g^−1^ at 0.5C, 1C, 5C, 10C, and 20C, respectively, significantly outperforming the cells using CCE and WCE (Fig. 4c and S16†). This indicates that, under these test conditions, the rate-limiting step for the Li-NCM811 cell to achieve high-rate charge–discharge is the Li^+^ transport and de-solvation capability at the interface rather than the ionic conductivity of the bulk electrolyte. Furthermore, long-term cycling testing at 5C revealed that SCE-based Li-NCM811 cells retained 76.2% of their initial capacity (157.6 mA h g^−1^) after more than 1000 cycles, whereas CCE-based cells retained only 25.7% after 500 cycles (Fig. 4d and S17†). The rapid failure of WCE-based cells after the initial cycle further underscores the limitations of this electrolyte at high voltages. Analysis of the d*Q*/d*V* curve from the initial charge–discharge cycle reveals that the Li-NCM811 cell with the SCE exhibits a more pronounced H1 + M peak intensity, reflecting faster Li^+^ extraction kinetics (Fig. S18†). Besides, the potential difference between the H2 + H3 reversible redox peaks is the smallest among the three electrolytes, measuring only 20.0 mV, compared to 42.8 mV for CCE and 36.6 mV for WCE. This suggests that the SCE helps mitigate polarization effects in the NCM811 electrode at high voltage.^50^ Moreover, control experiments showed that the cells based on dual-salt systems exhibited better rate performance and long-cycle stability compared to single-salt systems, indicating that the unique synergistic effect of LiNO_3_ and LiPO_2_F_2_ can jointly improve the electrochemical performance of Li-NCM811 cells, which may be related to the composition of the interface derived from them (Fig. S19 and S20†). Similar to the test results of the Li–Cu half-cell, the Li-NCM811 cell also shows the best cycle stability when the concentration ratio of LiNO_3_ and LiPO_2_F_2_ is 1 : 1 (Fig. S21†). This is because Li_3_N, produced by the decomposition of LiNO_3_, can effectively protect the LMA. Moreover, conductivity results indicate that LiNO_3_ is crucial for maintaining the electrolyte's conductivity. LSV results show that LiF and Li_3_PO_4_, generated from the decomposition of LiPO_2_F_2_, can effectively protect the NCM811 cathode, thereby ensuring the high-voltage stability of the electrolyte (Fig. S22†). Therefore, the optimal ratio of the two salts was determined to be 1 : 1. Long-cycle testing also confirmed that the optimal FEC additive amount for Li-NCM811 cells remained at 20 vol%, consistent with previous results from Li–Cu half-cell tests (Fig. S23†). Furthermore, even under low-temperature conditions (−20 °C), Li-NCM811 cells using SCE exhibited the best rate performance and long-term cycling stability, achieving stable cycling for more than 800 cycles at 1C, with a discharge capacity exceeding 90 mA h g^−1^ and a capacity retention rate of 89.4% (Fig. 4e, S24 and S25†). In contrast, CCE and WCE-based cells failed rapidly under low-temperature conditions. EIS data confirmed that the interface derived from the SCE exhibited the lowest interfacial resistance after the initial cycle, originating from the decomposition of anions, thus ensuring efficient Li^+^ transport (Fig. S26†).^51^

**Fig. 5:**
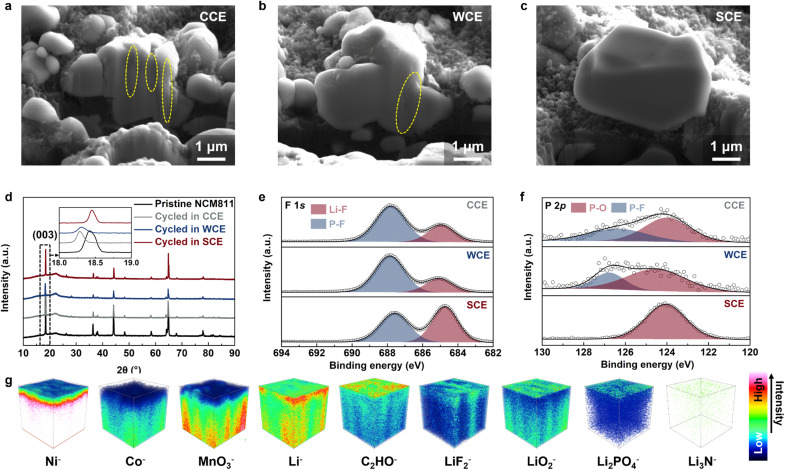
FIB-SEM images of cycled NCM811 cathodes using different electrolytes: (a) CCE, (b) WCE, (c) SCE. (d) XRD patterns of pristine and cycled NCM811 cathodes in the studied electrolytes. The inset shows a locally amplified XRD pattern. (e) The F 1s spectra and (f) P 2p spectra comparison of the cycled NCM811 cathodes in different electrolytes. (g) 3D views of the CEI composition detected by TOF-SIMS depth sputtering on the surface of cycled NCM811 using SCE.

Failure analysis of the NCM811 cathode after cycling demonstrates the significant impact of different electrolytes on its structural integrity. Focused ion beam scanning electron microscopy (FIB-SEM) results reveal substantial cracks within the cathode cycled in both CCE and WCE, indicating irreversible structural damage (Fig. 5a, b, S27 and S28†).^52^ In contrast, the NCM811 cathode cycled in SCE retained a smooth internal structure, suggesting that SCE offers superior protection for the cathode material (Fig. 5c and S29†). This observation is further supported by X-ray diffraction (XRD) analysis, which shows that the cathode cycled in SCE retained the highest peak intensity and exhibited the smallest shift in the (003) characteristic peak compared to those cycled in CCE and WCE (Fig. 5d).^53^ These results suggest that the formation of a stable CEI in SCE is crucial for preserving the cathode's structural integrity. Raman spectroscopy was used to examine the electrolyte before and after cycling to assess compositional changes. The results showed substantial decomposition of both CCE and WCE after cycling. Specifically, in CCE, there was a noticeable reduction in the intensities of the LiPF_6_ and EC peaks, while in WCE, the intensities of the peaks corresponding to LiPF_6_, LiFSI, and free DME also declined (Fig. S30 and S31†). However, no significant decrease in the signals of salts and solvents was observed in SCE, indicating the formation of a stable interfacial layer that prevents continuous electrolyte degradation (Fig. S32†). XPS results show that, similar to the composition of the SEI, the CEI formed in SCE contains more inorganic components and has a lower carbon content compared to the CEIs formed in CCE and WCE (Fig. S33†). The XPS F 1s and P 2p spectra further reveal that the CEI derived from SCE contains more LiF and Li_2_PO_4_ than those derived from CCE and WCE (Fig. 5e and f). Additionally, the XPS N 1s spectrum reveals the presence of Li_3_N in the CEI formed in SCE (Fig. S34†). The small signals corresponding to NO_2_^−^ and NO_3_^−^ are attributed to LiNO_2_ and residual LiNO_3_ at the interface, respectively. In SCE, LiF primarily originates from the decomposition of FEC and LiPO_2_F_2_ (Fig. S35†).^54,55^ Additionally, the decomposition of LiPO_2_F_2_ also generates Li_3_PO_4_, while Li_3_N forms from the decomposition of LiNO_3_.^56,57^ To further characterize the CEI structure formed on the NCM811 cathode after cycling in SCE, we performed time-of-flight secondary ion mass spectrometry (TOF-SIMS) analysis.^58^ The distribution of Ni^−^, Co^−^, and MnO_3_^−^ ions primarily within the inner NCM811 suggests that the NCM811 cathode was effectively protected, without significant dissolution of transition metals (Fig. 5g and S36†). The distribution of Li^−^ secondary ions indicates that the electrode surface is covered with a CEI rich in inorganic salts. The distribution of C_2_HO^−^ ions further suggests that the outermost layer of the CEI contains a large amount of organic components, which may result from the self-polymerization reaction after FEC defluorination. Specifically, the distributions of LiF_2_^−^, LiO_2_^−^, Li_2_PO_4_^−^, and Li_3_N^−^ indicate that the inorganic components in this CEI include a large amount of LiF, Li_2_O and Li_2_PO_4_, as well as a small amount of Li_3_N (Fig. S37†). LiF and Li_2_PO_4_ originate from the decomposition of LiPO_2_F_2_ and play a role in maintaining the structural stability of the cathode, while Li_2_O and Li_3_N mainly originate from the decomposition of LiNO_3_, which facilitates the transport of Li^+^. The intrinsic strong Coulomb interactions of LiNO_3_ and LiPO_2_F_2_, along with the synergistic effects of the aforementioned interface components, endow LMBs with excellent high-voltage stability and outstanding electrochemical performance.

**Fig. 6:**
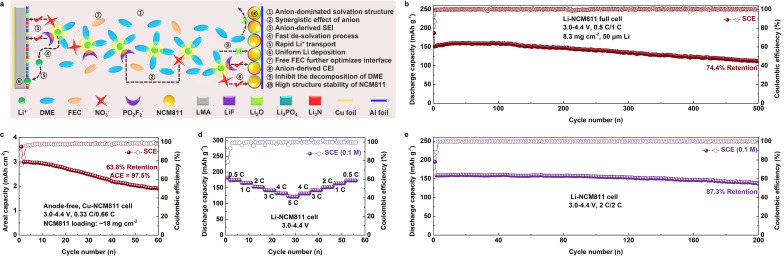
Schematic diagram of the effect mechanism of SCE on high-voltage LMBs. (b) Long-term cycling performance of the Li-NCM811 full cell and (c) Cu-NCM811 anode-free cell using SCE (test conditions are 8.3 mg cm^−2^, 50 μm Li, 0.5C/1C, and 18 mg cm^−2^, 0.33C/0.66C, respectively; 3.0–4.4 V, 1C = 180 mA g^−1^). (d) Rate and (e) long-term cycling performance of the Li-NCM811 cell using SCE with a salt concentration of 0.1 M. (SCE composition is 0.05 M LiNO_3_ + 0.05 M LiPO_2_F_2_ in DME/FEC (8 : 2, v/v); test conditions are 0.5C, 1C, 2C, 3C, 4C, 5C, and 2C/2C, respectively; 3.0–4.4 V, 1C = 180 mA g^−1^).

Based on the characterization and testing results, we propose the mechanism by which SCE affects high-voltage LMBs, as illustrated in Fig. 6a. The SCE, characterized by strong Coulomb interactions, forms an anion-dominant solvation structure. This solvation structure not only accelerates the de-solvation process of Li^+^ but also facilitates the formation of stable interfacial protection layers at both the cathode and anode, promoting fast Li^+^ transport and inhibiting the decomposition of DME. Consequently, these effects enable stable cycling and fast charge–discharge in high-voltage LMBs. Our designed SCE fundamentally differs from traditional WSE and HCE in its approach to solvation structure regulation. SCE leverages low-dissociation salts to tailor solvation without being confined to a specific solvent or concentration. In contrast, WSE modifies solvation by reducing the solvent's coordinating ability, whereas HCE increases salt concentration to lower the free solvent content, thereby enhancing anion participation in the inner solvation shell. Moreover, WSE and HCE typically employ highly dissociative LiFSI, which often corrodes the current collector.^59^ In contrast, SCE avoids the use of LiFSI, thereby exhibiting excellent high-voltage stability. To further verify the practicality of SCE, we assembled Li-NCM811 full cells under practical conditions. With a cathode loading of 8.3 mg cm^−2^ and thin Li foil (50 μm), the Li-NCM811 full cell based on SCE demonstrated stable cycling for over 500 cycles, with a capacity retention of 74.4% (Fig. 6b and S38†). Despite the cathode loading being elevated to 18 mg cm^−2^ and the electrolyte volume reduced to 25 μL (lean electrolyte), the SCE-based Li-NCM811 full cell exhibits stable cycling behavior under harsh conditions, thereby validating the practical applicability of SCE in LMBs (Fig. S39†). Moreover, when a commercial NCM811 cathode (loading of 18 mg cm^−2^) was used to assemble an anode-free LMB, the Cu-NCM811 cell using SCE also exhibited stable cycling for over 60 cycles, with a capacity retention of 63.8% and an ACE of 97.5%, further indicating the promising application potential of SCE (Fig. 6c and S40†).^60^ Importantly, to demonstrate the universality of this strong Coulomb interaction electrolyte design principle, we further reduced the electrolyte concentration to an ultra-low level of 0.1 M, with a composition of 0.05 M LiNO_3_ + 0.05 M LiPO_2_F_2_ in DME/FEC (8 : 2, v/v), and evaluated its electrochemical performance in high-voltage LMBs. Surprisingly, even at this ultra-low concentration of 0.1 M, the SCE enabled the Li-NCM811 cell to maintain good rate performance at a cutoff voltage of 4.4 V, with a discharge-specific capacity of over 120 mA h g^−1^ at 5C (Fig. 6d and S41†). Additionally, the Li-NCM811 cell using SCE also exhibited excellent cycling stability, maintaining stable performance for more than 200 cycles under 2C charge–discharge conditions, with a capacity retention of 87.3% (Fig. 6e and S42†). The superior electrochemical performance of ultra-low concentration SCE highlights its significant advantages over previously reported low-concentration electrolytes (Table S1†).

## Conclusions

In conclusion, we present a novel strategy for designing high-voltage electrolytes by adjusting the interaction strengths among Li^+^, anions, and solvent molecules. The strong Coulomb interaction between LiNO_3_ and LiPO_2_F_2_ facilitates the creation of an anion-dominated solvation structure, which enables DME-based electrolytes to maintain excellent high-voltage stability (4.4 V) at an ultra-low concentration of 0.1 M. The unique synergistic effects of LiNO_3_ and LiPO_2_F_2_ result in the formation of an inorganic interface protection layer, consisting of LiF, Li_3_PO_4_, and Li_3_N, on both electrode surfaces. This protective layer is crucial for preserving electrode integrity and suppressing side reactions. Electrochemical performance tests show that Li-NCM811 cells with SCE deliver impressive rate capabilities (20C/120.8 mA h g^−1^) and long-term cycle stability (5C/1000 cycles). Even under practical conditions, the Li-NCM811 full cell using SCE demonstrates stable cycling for over 500 cycles. This work not only presents a novel approach to high-voltage electrolyte design but also offers a high-performance, low-cost electrolyte for practical LMBs.

## Data availability

Data are available from the authors on reasonable request.

## Author contributions

Z. J. and C. L. conceived and designed this work. C. L. and Z. J. carried out the synthesis, electrochemical measurements and computational calculations. Z. J., Y. Z., W. X., J. Z., S. W., M. S. and Y. L. participated in the analysis of the data. All authors discussed and revised the manuscript.

## Conflicts of interest

There are no conflicts to declare.

## Supplementary Material

SC-OLF-D4SC07393B-s001
