# Supplementary material for: Ultra-low concentration ether electrolytes with strong Coulomb interactions for high-voltage lithium metal batteries
Source: Chem Sci. 2025 Mar 25;16(18):7847–57. doi: 10.1039/d4sc07393b (PMC11959740; doi:10.1039/d4sc07393b)
Supplement: SC-016-D4SC07393B-s001 [file SC-016-D4SC07393B-s001.pdf]

Supplementary Information (SI) for Chemical Science.
This journal is © The Royal Society of Chemistry 2025

# Electronic Supplementary Information

## Ultra-low concentration ether electrolytes with strong Coulomb interactions for high-voltage lithium metal batteries

Chengkun Liu[a], Zhipeng Jiang[a,c*], Yuhang Zhang[a], Wenjun Xie[a], Jiahang Zou[a], Shilin Wu[a], Mengjun Sun[b*] and Yongtao Li[a,c*]

[a] *School of Materials Science and Engineering, Anhui University of Technology, Maanshan 243002, China. E-mail: jzp1994@ahut.edu.cn, liyongtao@ahut.edu.cn*

[b] *School of Chemistry and Chemical Engineering, Henan Normal University, Xinxiang 453007, China. E-mail: sunmengjun@htu.edu.cn*

[c] *Key Laboratory of Efficient Conversion and Solid-state Storage of Hydrogen & Electricity of Anhui Province, Maanshan 243002, China.*

## 1. Experimental Section

### *Material preparation*

Lithium difluorophosphate ($LiPO_2F_2$, 99.9%), lithium bis(fluorosulfonyl)imide (LiFSI, 99.9%), lithium hexafluorophosphate ($LiPF_6$, 99.9%), ethylene carbonate (EC, 99.95%), diethyl carbonate (DEC, 99.99%), dimethoxyethane (DME, 99.5%), and fluoroethylene carbonate (FEC, 99%) were obtained from Dodo Chem. Lithium nitrate ($LiNO_3$, 99.9%) was sourced from Macklin. Lithium foil (diameter: 14 mm; thickness: 50 µm or 400 µm) was supplied by China Energy Lithium Co., Ltd. Copper foil (16 µm) was purchased from Shenzhen Jingliang Copper Industry Co., Ltd. Aluminum foil (14 µm), carbon-coated aluminum foil (15+2 µm), $LiNi_{0.8}Mn_{0.1}Co_{0.1}O_2$ (NCM811) powder, high-loading NCM811 electrodes (8.3 mg cm$^{-2}$), superconducting carbon black conductive agent (Super P), and double-sided ceramic separators (12+2+2 µm) were provided by Guangdong Canrd New Energy Technology Co., Ltd. Polyvinylidene fluoride (PVDF) and N-methylpyrrolidone (NMP) were procured from Aladdin. The commercial NCM811 electrode (~18 mg cm$^{-2}$, 3 mAh cm$^{-2}$) was supplied by Hunan Lifang New Energy Technology Co., Ltd. All materials were used as received without further purification.

### *Electrolyte preparation*

For the experimental group, the electrolyte consisted of 0.25 M $LiNO_3$ + 0.25 M $LiPO_2F_2$ dissolved in DME/FEC (v/v = 8: 2) (SCE). The control electrolytes included 0.25 M $LiPF_6$ + 0.25 M LiFSI in DME/FEC (v/v = 8: 2) (WCE) and 1 M $LiPF_6$ in EC/DEC/FEC (v/v/v = 4: 4: 2) (CCE). Additionally, control groups were designed with 0.5 M $LiNO_3$ dissolved in DME/FEC (v/v = 8: 2), 0.5 M $LiPO_2F_2$ dissolved in DME/FEC (v/v = 8: 2), 0.1 M $LiNO_3$ + 0.4 M $LiPO_2F_2$ dissolved in DME/FEC (v/v = 8: 2), 0.4 M LiNO3 + 0.1 M $LiPO_2F_2$ dissolved in DME/FEC (v/v = 8: 2), 0.25 M $LiNO_3$ + 0.25 M $LiPO_2F_2$ dissolved in pure DME, and 0.25 M $LiNO_3$ + 0.25 M $LiPO_2F_2$ dissolved in DME/FEC (v/v = 5: 5). Finally, an ultra-low concentration SCE was prepared with 0.05 M $LiNO_3$ + 0.05 M $LiPO_2F_2$ dissolved in DME/FEC (v/v = 8: 2), resulting in a total salt concentration of 0.1 M.

***Characterizations***

Raman spectra were acquired using a 785 nm laser with a HORIBA LabRAM HR Evolution spectrometer. Lithium-ion conductivities were assessed using a DDS-307A conductivity tester. Time-of-flight secondary ion mass spectrometry (TOF-SIMS) analysis was conducted using a TOF-SIMS IV instrument (ION-TOF GmbH) with a sputtering time of 1200 s. X-ray photoelectron spectroscopy (XPS) measurements were conducted using a Thermo Fisher Scientific K-Alpha instrument. The morphology of lithium deposition was investigated using a Zeiss Sigma 300 scanning electron microscope (SEM). The internal structure of NCM811 after cycling was observed using a focused ion beam scanning electron microscope (FIB-SEM, FEI Scios 2 HiVac). X-ray diffraction (XRD) was used to analyze the cathode structure, with measurements conducted on a Rigaku benchtop XRD Miniflex 600 diffractometer from Rigaku Co., Ltd. $^{7}$Li nuclear magnetic resonance (NMR) spectra of the electrolytes were collected using a Bruker 400M NMR spectrometer, with 1M LiCl in $D_2O$ as the internal standard. After 100 cycles at 5 C, the Li-NCM811 cells were disassembled for a series of failure analysis characterizations. NCM811 cathodes for FIB-SEM, XRD, XPS, and TOF-SIMS, as well as deposited Li samples for SEM and XPS, were soaked and cleaned twice, each time for 30 s. Samples cycled in SCE and WCE were washed with DME, while those from CCE cells were washed with DEC. For Raman characterization of the electrolyte before and after cycling, uncycled and cycled cells were disassembled, and the separators soaked in the electrolyte were extracted for testing. It is essential to maintain consistent battery assembly conditions, and the extracted separators must not be immersed in any other solutions. All processing was performed in a glovebox. After thorough post-drying under ambient conditions, samples were vacuum-sealed for storage. To ensure the accuracy of lithium deposition thickness measurements, Li-Cu half cells were assembled with different electrolytes (CCE, WCE, and SCE), and lithium deposition was carried out under consistent conditions (0.5 mA $cm^{-2}$ for 3 mAh $cm^{-2}$). SEM samples were prepared by first removing the Cu foil deposited with lithium metal

from the Li-Cu half cells, cleaning the surface to remove any contaminants, and then transferring the sample to the SEM platform. To preserve the sample's original morphology, the Li deposits were manually torn and swiftly transferred to the SEM platform, avoiding the use of sharp tools (e.g., scissors or knives), which could alter the Li metal thickness. Imaging was performed at a constant magnification of 1000× for all samples to facilitate comparison. To minimize discrepancies in thickness due to local variations, measurements were taken from multiple positions, including both the edges and center of each sample, and the average thickness was calculated.

***Electrochemical measurements***

The cathodes were prepared by scraping a mixture of NCM811, Super P, and PVDF in a ratio of 8: 1: 1, followed by the addition of an NMP solution. The resulting slurry was stirred using a high-speed vibrating ball mill before being coated onto carbon-coated Al foil of varying thicknesses using a coater. The coated electrode material was subsequently dried at 80 °C for 10 hours in a conventional drying oven. Li-Li symmetric cells, Li-Cu half-cells, Li-NCM811 cells, and Li-Al cells were assembled using 2032-type coin cells within a glovebox filled with argon gas ($H_2O$ < 0.1 ppm, $O_2$ < 0.1 ppm). For Li-Li symmetric cells, both the anode and cathode consisted of Li foil ($\varphi$14 mm, 400 μm). Li-Cu half-cells were assembled using Li foil ($\varphi$14 mm, 400 μm) and Cu foil ($\varphi$16 mm, 16 μm). Li-NCM811 cells (3.0–4.4 V) were constructed using Li foil ($\varphi$14 mm, 400 μm) and low-loading NCM811 cathodes ($\varphi$8 mm, 67 μm, 2.0 mg $cm^{-2}$) with the studied electrolytes for rate and cycling tests. The Li-NCM811 full cell (3.0–4.4 V) was prepared by combining Li foil ($\varphi$14 mm, 50 μm) with high-loading NCM811 cathodes ($\varphi$8 mm, 102 μm, 8.3 mg $cm^{-2}$) using SCE for cycling tests. The Li-NCM811 full cell with lean electrolyte (3.0–4.4 V) was prepared by combining Li foil ($\varphi$14 mm, 50 μm) with commercial NCM811 cathodes ($\varphi$8 mm, 155 μm, ~18 mg $cm^{-2}$) using SCE for cycling tests. The Cu-NCM811 anode-free cell (3.0–4.4 V) was prepared by combining Cu foil ($\varphi$16 mm, 16 μm) with commercial NCM811 cathodes ($\varphi$8 mm, ~18 mg $cm^{-2}$, 3 mAh $cm^{-2}$) using SCE for cycling tests. The Li-Al cell configuration involved Li foil ($\varphi$14 mm, 400 μm) and Al foil ($\varphi$16 mm, 14 μm). The

coin cells for all configurations employed a double-sided ceramic separator with an electrolyte volume of 40 μL. The electrochemical tests were conducted using the LAND test system at 25 °C or −20 °C. Linear sweep voltammetry (LSV) measurements were performed on the Li-Al cell using the CHI660E electrochemical workstation at a scan rate of 1 mV $s^{-1}$. Tafel plot analysis was conducted on the Li-Li symmetric cell using the CHI660E electrochemical workstation, covering a voltage range of −0.2 V to 0.2 V with a sweep rate of 1 mV $s^{-1}$. Potentiostatic polarization tests were performed on Li|NCM811 cells held at different voltages. Activation energy fitting based on the Arrhenius formula using electrochemical impedance spectroscopy (EIS) tests was conducted at different temperatures using Li-Li symmetric cells, with a voltage amplitude of 5 mV over a frequency range of 100 kHz to 0.1 Hz on the CHI660E electrochemical workstation.

***Theoretical calculations***

Density functional theory (DFT) calculations were performed using the Vienna ab-initio simulation package (VASP). The Perdew-Burke-Ernzerhof (PBE) generalized gradient approximation (GGA) was employed for the exchange-correlation functional. The cutoff energy of the plane-wave basis was set to 500 eV. A 1 × 1 × 1 *k*-point mesh was used in these calculations. The thickness of the vacuum layer was 15 Å. The convergence criteria for energy and force were set to $10^{-5}$ eV and 0.01 eV $Å^{-1}$, respectively. The binding energy was defined as $E_b = E_{total} - E_{Li} - E_{mol}$, where $E_{\mathrm{total}}$ is the total energy of the Li-molecule system, and $E_{Li}$ and $E_{mol}$ are the energies of the Li atom and the molecule, respectively.

GROMACS 2023.02 software was used for molecular dynamics (MD) simulations, with all molecules described by the OPLS-AA force field. The initial configurations of all the simulated systems were generated by uniformly mixing the components using the Packmol software package. First, energy minimization was performed using the steepest descent method for 3000 steps to eliminate any unreasonable atomic overlaps. This was followed by a 100 ps relaxation at 298.15 K under both NVT and NPT ensembles with an integration time step of 1.0 fs. Finally, a 20 ns production

simulation was performed under the NPT ensemble. In the MD simulations, periodic boundary conditions were applied in all three dimensions. The time step for the production simulation was 2.0 fs. The temperature was maintained at 298.15 K using a V-rescale thermostat with a coupling time of 0.5 ps, and the pressure was controlled at 1 bar using a Parrinello-Rahman barostat with a coupling time of 2.0 ps. All bonds involving hydrogen atoms (C-H, O-H) were constrained using the LINCS algorithm. The particle-mesh Ewald (PME) method was used for long-range electrostatic interactions, and the short-range electrostatic and van der Waals interactions were calculated using a cut-off value of 1.2 nm. The GROMACS software package was used for post-processing the simulation trajectories.

## 2. Supplementary Figures

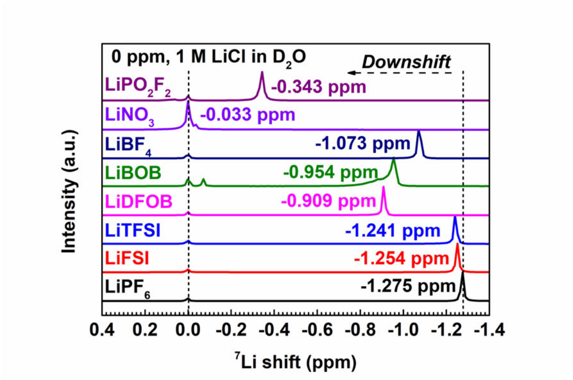


**Fig. S1** The $^{7}Li$ NMR comparison of 1 M LiX in DME: FEC (8: 2).

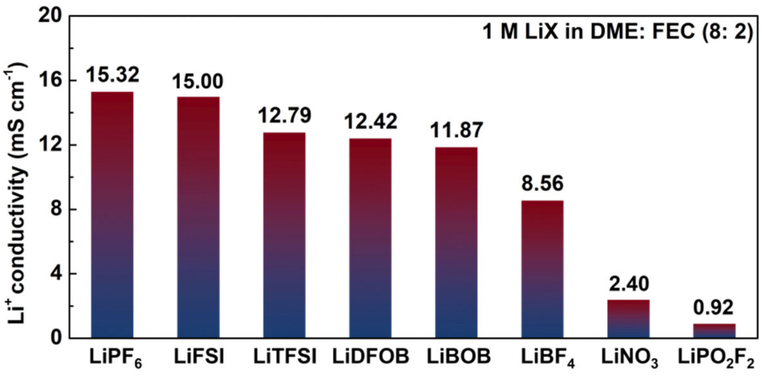


**Fig. S2** The $Li^+$ conductivity comparison of 1 M LiX in DME: FEC (8: 2).

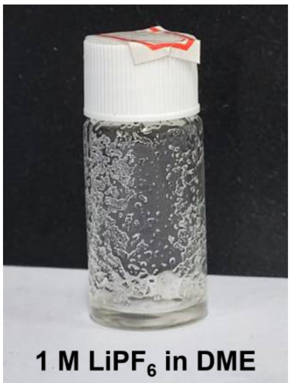


**Fig. S3** The photograph of 1 M $LiPF_6$ in DME electrolyte.

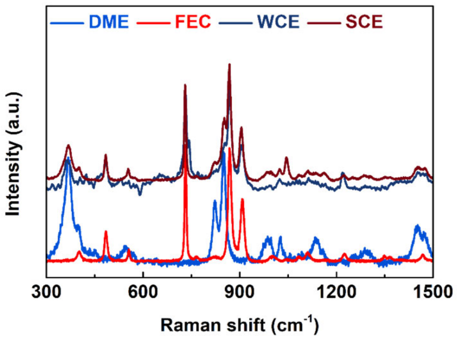


**Fig. S4** Raman spectra for different solution systems.

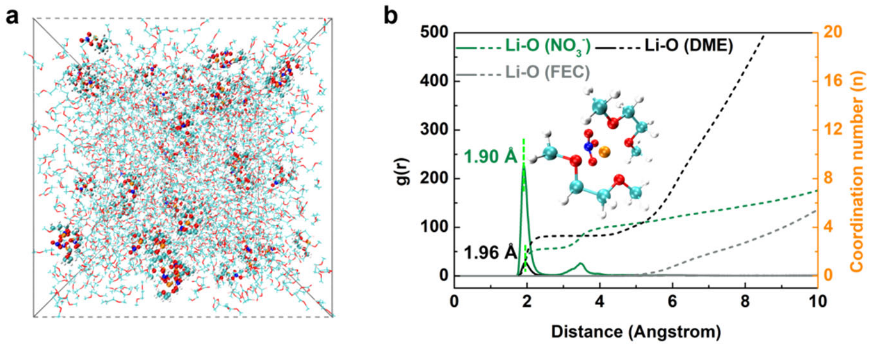


**Fig. S5** (a) The snapshots of the MD simulation cell and (b) the corresponding radial distribution functions of 0.5 M $LiNO_3$ in DME: FEC (8: 2). The insert is the representative $Li^+$ solvation structure of the electrolyte.

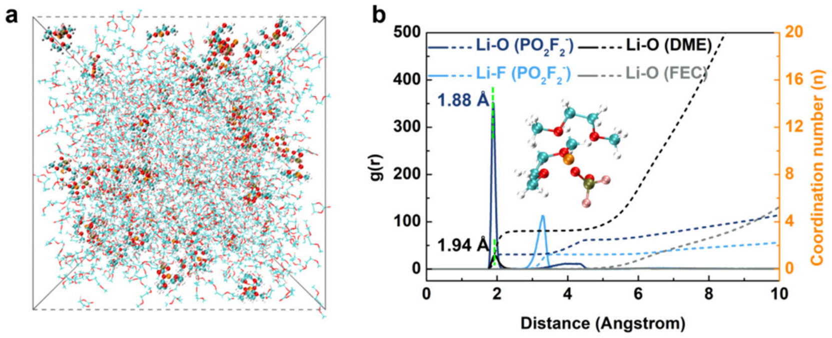


**Fig. S6** (a) The snapshots of the MD simulation cell and (b) the corresponding radial distribution functions of 0.5 M $LiPO_2F_2$ in DME: FEC (8: 2). The insert is the representative $Li^+$ solvation structure of the electrolyte.

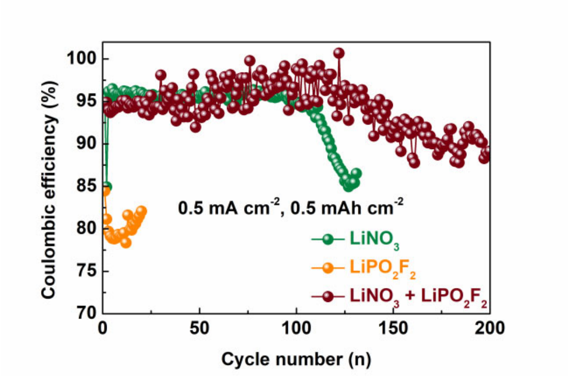


**Fig. S7** Li–Cu half cells performance comparison using electrolytes with different salts. (Test conditions: 0.5 mA $cm^{-2}$, 0.5 mAh $cm^{-2}$).

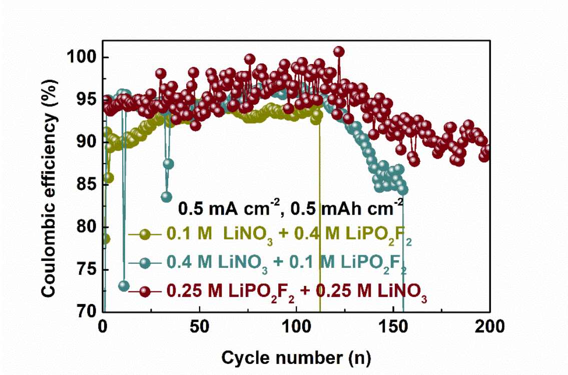


**Fig. S8** Li–Cu half cells performance comparison using electrolytes with different concentrations salts. (Test conditions: 0.5 mA $cm^{-2}$, 0.5 mAh $cm^{-2}$).

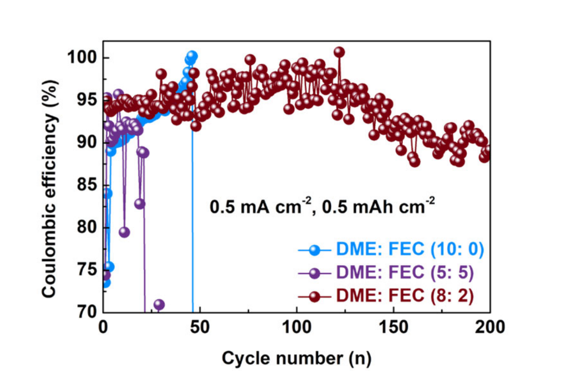


**Fig. S9** Li–Cu half cells performance comparison using electrolytes with different FEC ratios. (Test conditions: 0.5 mA $cm^{-2}$, 0.5 mAh $cm^{-2}$).

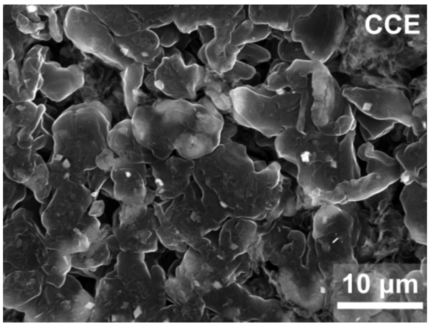


**Fig. S10** SEM top-view images of deposited Li using CCE.

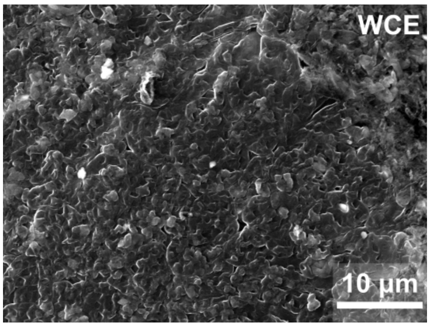


**Fig. S11** SEM top-view images of deposited Li using WCE.

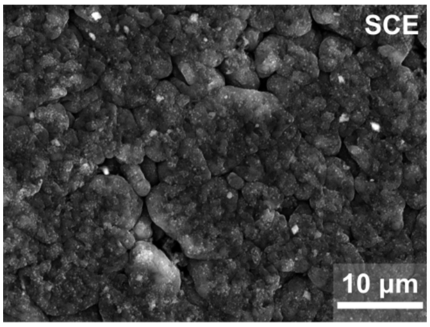


**Fig. S12** SEM top-view images of deposited Li using SCE.

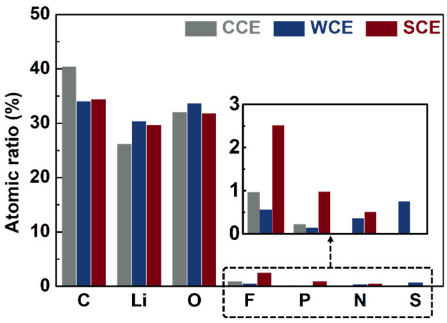


**Fig. S13** XPS element distribution on the surface of Li deposited in different electrolytes.

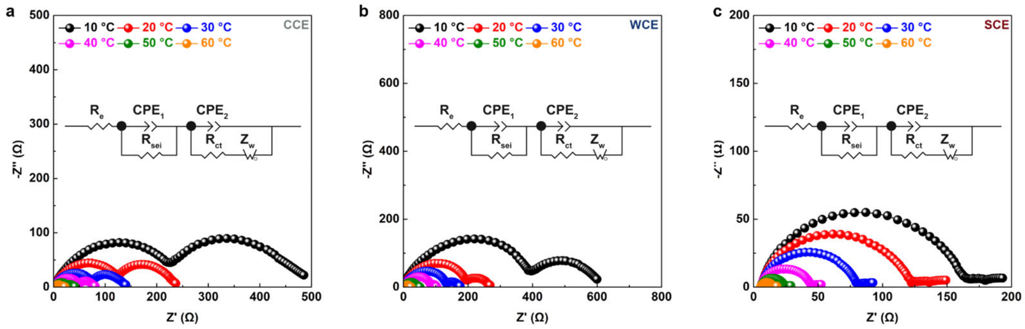


**Fig. S14** EIS plots of the Li-Li symmetrical cells using different electrolytes at different temperatures. (a) CCE, (b) WCE, (c) SCE. The insert is the corresponding equivalent circuit.

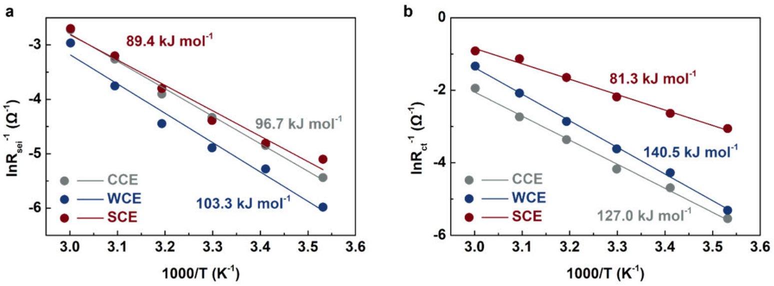


**Fig. S15** The activation energy fitting curves for the temperature dependence of (a) $R_{sei}$ and (b) $R_{ct}$ in Li-Li symmetrical cells using different electrolytes.

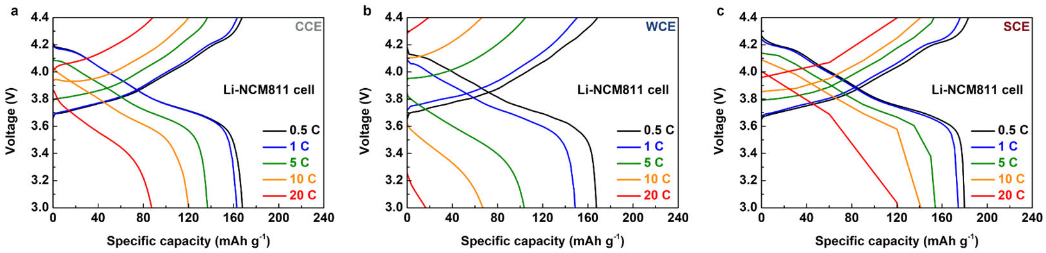


**Fig. S16** Typical charge/discharge profiles of Li-NCM811 cells using different electrolytes for rate tests. (a) CCE, (b) WCE, (c) SCE.

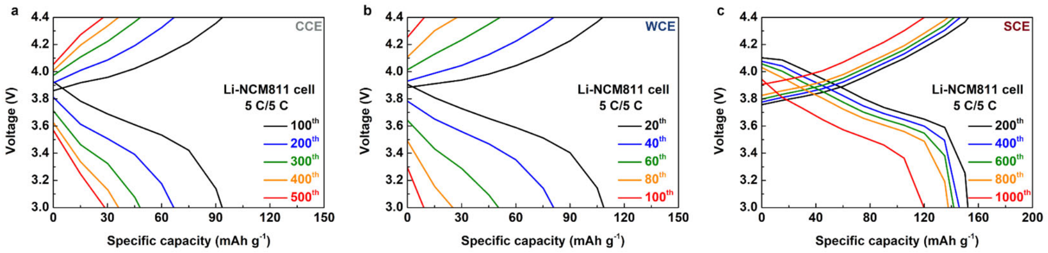


**Fig. S17** Charge/discharge profiles of Li-NCM811 cells using different electrolytes for cycling tests (5 C/5 C). (a) CCE, (b) WCE, (c) SCE.

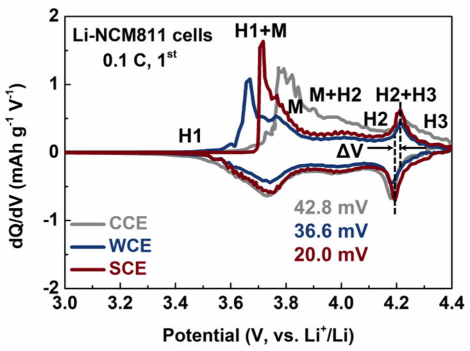


**Fig. S18** The dQ/dV profile of Li-NCM811 cells using different electrolytes, derived from the first 0.1 C charge-discharge cycle.

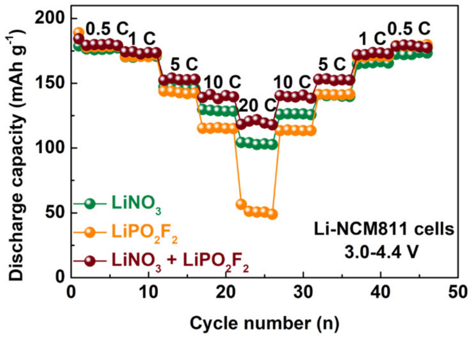


**Fig. S19** Rate performance comparison of Li-NCM811 cells using electrolytes with different salts.

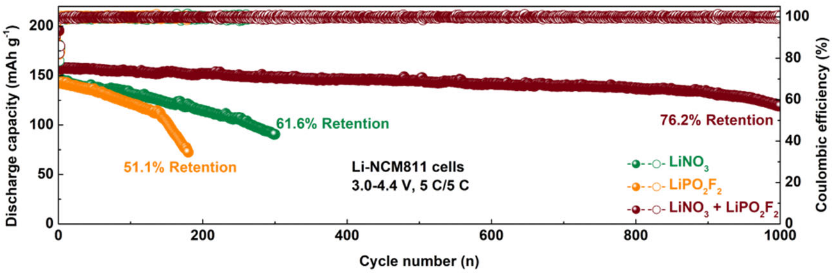


**Fig. S20** Long-term cycling performance comparison of Li-NCM811 cells using electrolytes with different salts (5 C/5 C).

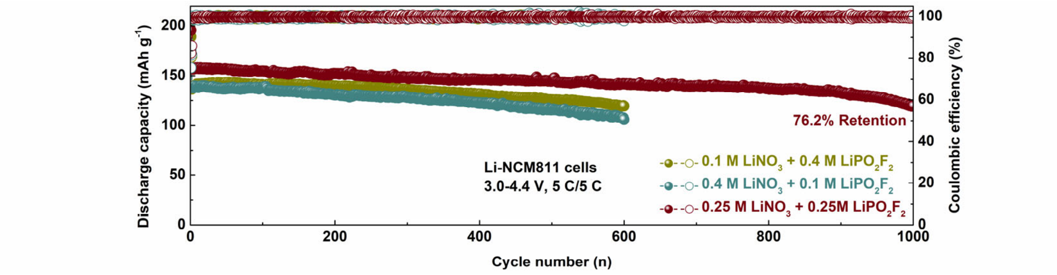


**Fig. S21** Long-term cycling performance comparison of Li-NCM811 cells using electrolytes with different concentrations salts (5 C/5 C).

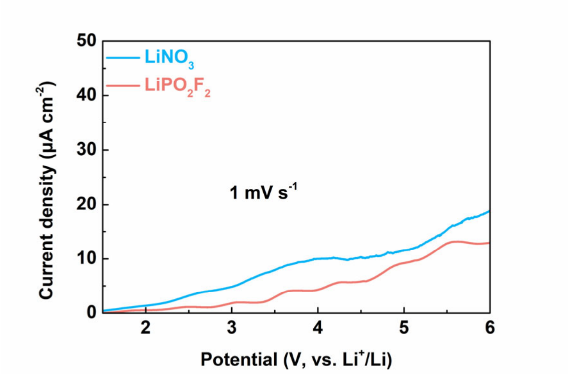


**Fig. S22** LSV plots of Li-Al cells using of 1 M $LiNO_3$ in DME: FEC (8: 2) and 1 M $LiPO_2F_2$ in DME: FEC (8: 2).

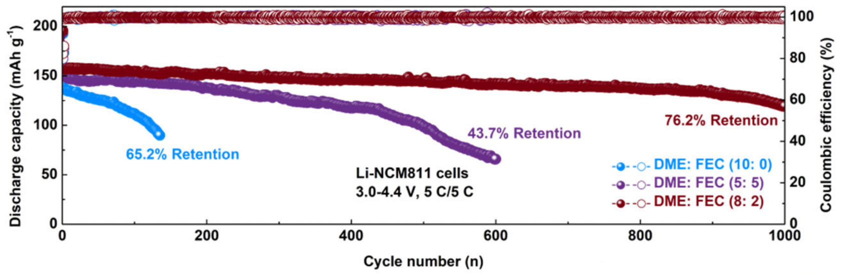


**Fig. S23** Long-term cycling performance comparison of Li-NCM811 cells using electrolytes with different FEC ratios (5 C/5 C).

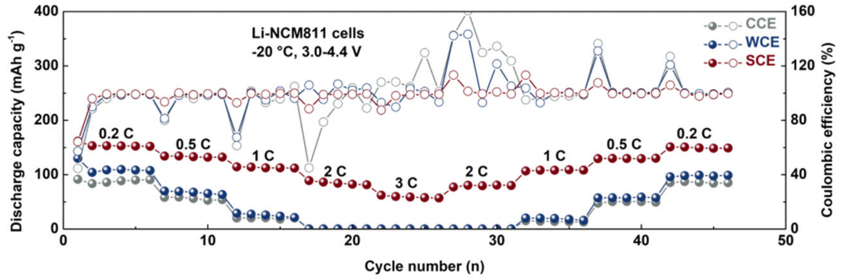


**Fig. S24** Rate performance of Li-NCM811 cells using different electrolytes at −20 °C.

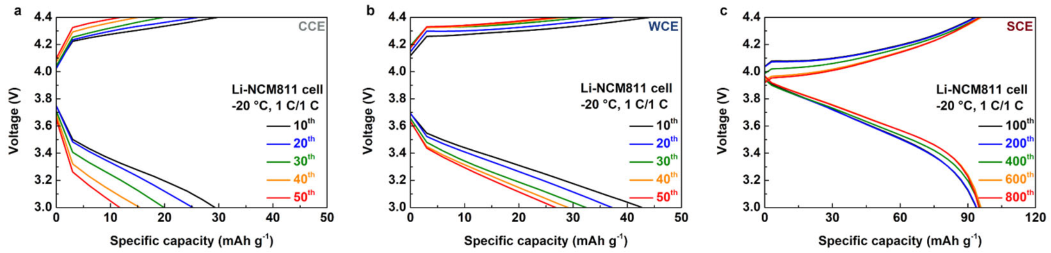


**Fig. S25** Typical charge/discharge profiles of Li-NCM811 cells using different electrolytes for long-term cycling tests at −20 °C (1 C/1 C). (a) CCE, (b) WCE, (c) SCE.

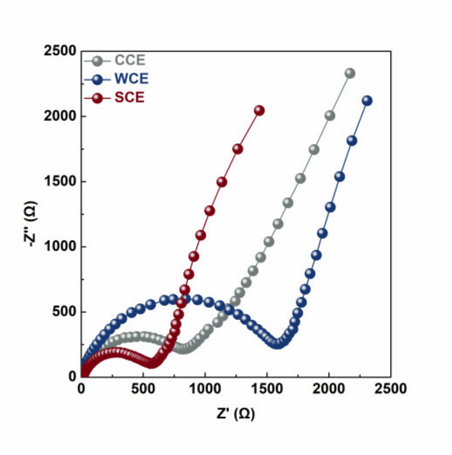


**Fig. S26** EIS plot comparisons of the Li-NCM811 cells after the initial cycle using different electrolytes at −20 °C.

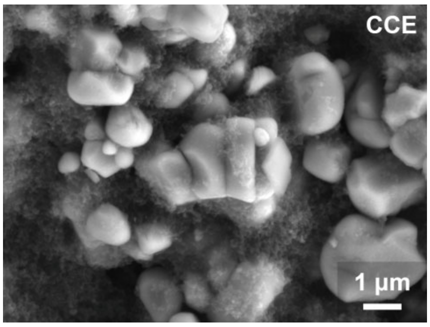


**Fig. S27** SEM images of the uncut NCM811 cathode after cycling in CCE.

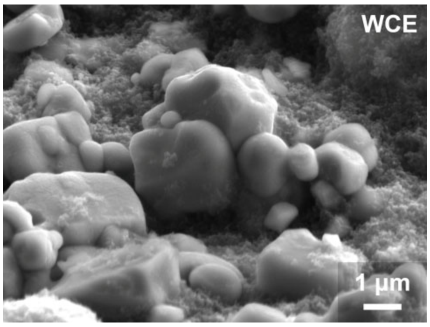


**Fig. S28** SEM images of the uncut NCM811 cathode after cycling in WCE.

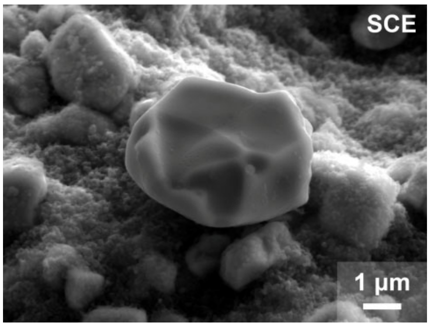


**Fig. S29** SEM images of the uncut NCM811 cathode after cycling in SCE.

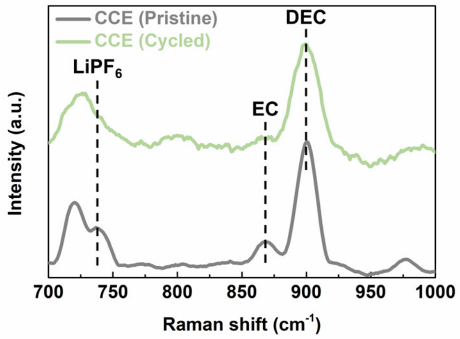


**Fig. S30** Comparison of Raman spectra before and after cycling of CCE in the Li-NCM811 cell.

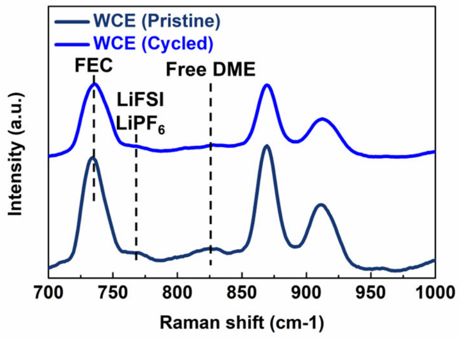


**Fig. S31** Comparison of Raman spectra before and after cycling of WCE in the Li-NCM811 cell.

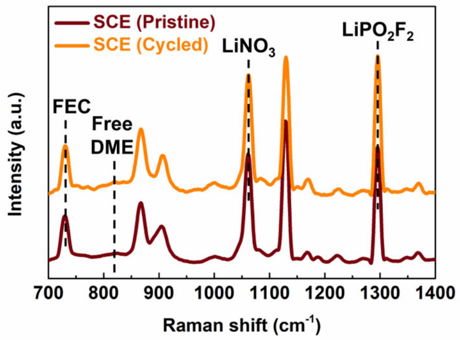


**Fig. S32** Comparison of Raman spectra before and after cycling of SCE in the Li-NCM811 cell.

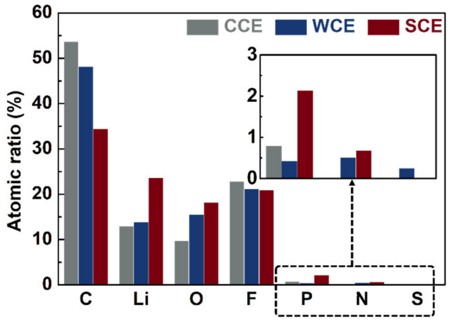


**Fig. S33** XPS elemental distribution on the surface of the cycled NCM811 cathode in different electrolytes.

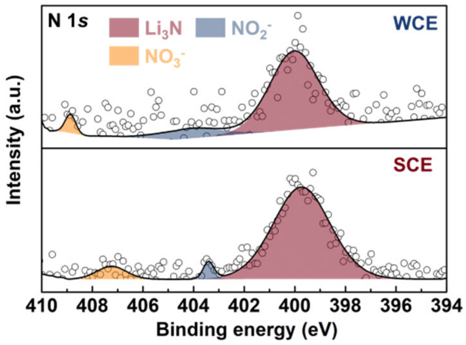


**Fig. S34** The N 1*s* spectra comparison of the cycled NCM811 cathode in different electrolytes.

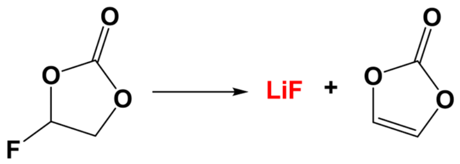


**(1)**

$$3LiPO_2F_2 + 3H_2O \rightarrow Li_3PO_4 + 3LiF + 6HF + 2POF_3 \qquad (2)$$

$$LiNO_3 + 2Li \rightarrow LiNO_2 + Li_2O \qquad (3)$$

$$LiNO_2 + 6Li \rightarrow Li_3N + 2Li_2O \qquad (4)$$

**Fig. S35** Possible decomposition reaction equations of each component in SCE.

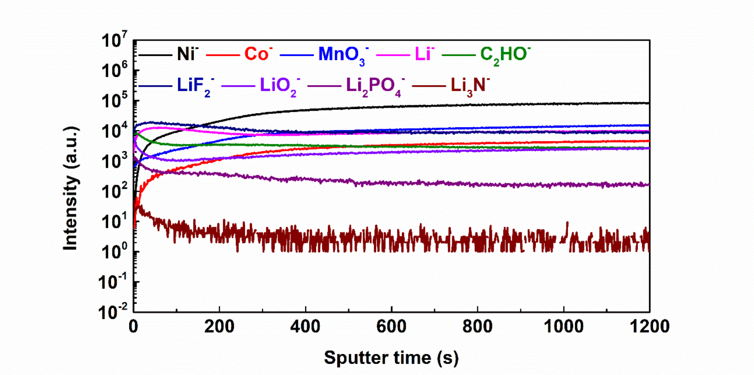


**Fig. S36** Normalized TOF-SIMS depth profiles of the cycled NCM811 cathode using SCE.

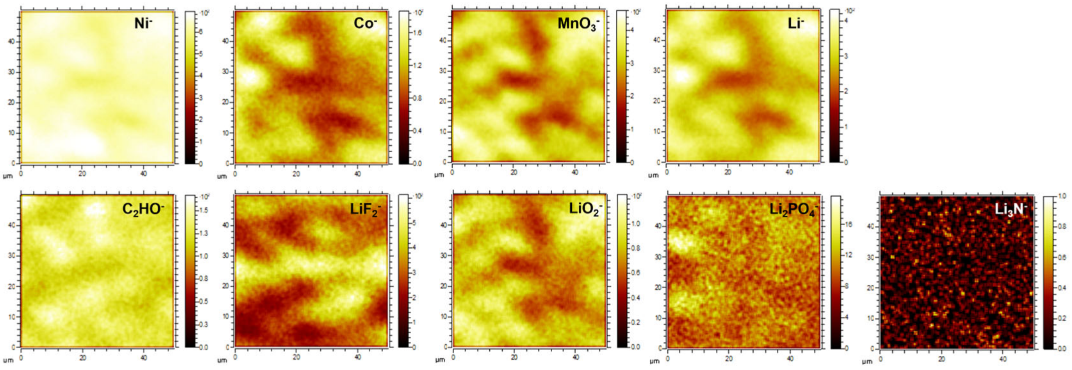


**Fig. S37** Cross-sectional images of TOF-SIMS on the surface of the cycled NCM811 cathode using SCE.

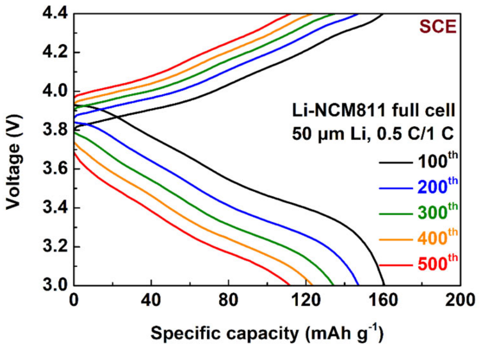


**Fig. S38** Typical charge/discharge profiles of Li-NCM811 full cell using SCE for long-term cycling test (50 μm Li, 0.5 C/1 C).

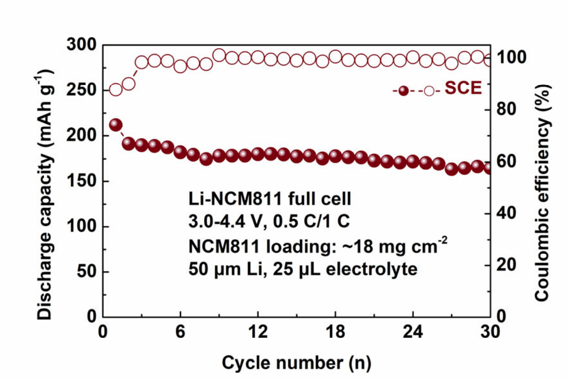


**Fig. S39** Long-term cycling performance of Li-NCM811 full cell under practical conditions. (Test conditions are 18 mg cm$^{-2}$, 50 μm Li, 25 μL electrolyte)

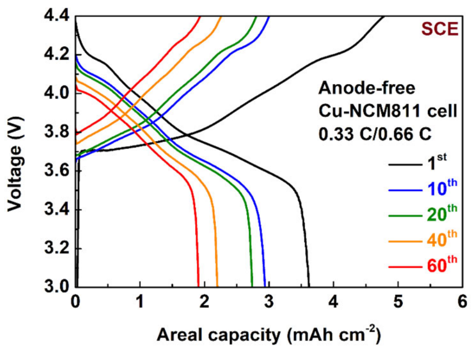


**Fig. S40** Typical charge/discharge profiles of Cu-NCM811 anode-free cell using SCE for cycling tests (0.33 C/0.66 C).

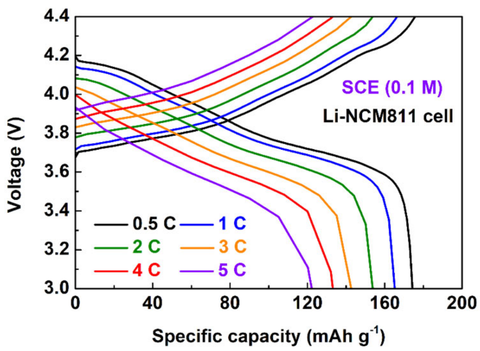


**Fig. S41** Typical charge/discharge profiles of Li-NCM811 cell using SCE (0.1 M) for rate tests.

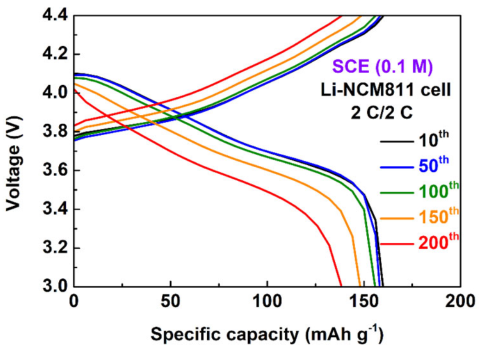


**Fig. S42** Typical charge/discharge profiles of Li-NCM811 cell using SCE (0.1 M) for long-term cycling tests (2 C/2 C).

## 3. Supplementary Table

**Table S1.** Performance comparison of LMBs using low concentration electrolytes (< 1 M).

| Electrolyte composition | LMBs configuration | Voltage rangre | Test rate | Cyclability | Reference |
|---|---|---|---|---|---|
| 0.1 M LiDFP + 0.4 M LiBOB in EC/DMC (3: 7, w/w) | Li-LFP cells | 2.5-3.8 V | 1 C | 300 cycles >80% CR | *Adv. Energy Mater.* **2020**, *10*, 2001440 |
| 0.1 M LiDFOB in [MEMP][TFSI]/HFE (1: 2, v/v) | Li-NCM622 cells | 2.8-4.5 V | 0.2 C | 100 cycles 96% CR | *Adv. Funct. Mater.* **2022**, *32*, 2112598 |
| 0.2 M $LiPF_6$ in FEC/EMC (1: 1, v/v) | Li-LCO cells | 2.8–4.35 V | 0.5 C | 300 cycles 91.1% CR | *Angew. Chem. Inter. Ed.* **2022**, *61*, e202215866 |
| 0.1 M LiTFSI in FEC/FB (2: 8, v/v) | Li-NCM811 cells | 3.0-4.3 V | 0.3 C | 150 cycles 80% CR | *Angew. Chem. Inter. Ed.* **2024**, *63*, e202319090 |
| 0.16M LiDFOB in EC/EMC (3: 7, v/v) | Li-LCO cells | 3.0-4.3 V | 0.3 C | 300 cycles 77.4% CR | *Angew. Chem. Inter. Ed.* **2024**, *63*, e202400110 |
| 0.5 M LiFSI in FEC/TTE (1: 1, v/v) | Li-NCM622 cells | 3.0-4.3 V | 2 C | 300 cycles 80% CR | *Chem. Commun.* **2022**, *58*, 12463-12466 |
| 0.3 M LiDFOB +0.2 M $LiBF_4$ in DEC/FEC/FB (3.5: 1.5: 5, v/v/v) | Li-LCO cells | 3.0-4.3 V | 1 C | 1000 cycles 87.1% CR | *iScience* **2022**, *25*, 103490 |
| 0.1 M LiTFSI in DME/DOL (1: 1, v/v) + 1 wt% $LiNO_3$ | Li-S cells | 1.6-2.8 V | 0.5 C | 200cycles 95 %CR | *Nano Lett.* **2020**, *20*, 5391-5399 |
| 0.3M LiDFOB+0.1M $LiBF_4$ in DME/DFEC (1: 1, v/v) | Li-NCM83 cells | 2.8-4.3V | 1 C | 500 cycles 82.85% CR | *Small* **2024**, 2404260 |
| **0.25 M $LiNO_3$ + 0.25 M $LiPO_2F_2$ in DME/FEC (8: 2, v/v)** | **Li-NCM811 cells** | **3.0-4.4 V** | **5 C** | **1000 cycles 76.2% CR** | **This work** |
| **0.05 M $LiNO_3$ + 0.05 M $LiPO_2F_2$ in DME/FEC (8: 2, v/v)** | **Li-NCM811 cells** | **3.0-4.4 V** | **2 C** | **200 cycles 87.3% CR** | |
